# Fundamental limits to multi-functional and tunable nanophotonic response

**DOI:** 10.1515/nanoph-2023-0630

**Published:** 2024-01-04

**Authors:** Hyungki Shim, Zeyu Kuang, Zin Lin, Owen D. Miller

**Affiliations:** Department of Applied Physics, Physics, and Energy Sciences Institute, Yale University, New Haven, CT 06511, USA; Bradley Department of Electrical and Computer Engineering, Virginia Tech, Blacksburg, VA, USA

**Keywords:** fundamental limits, multi-functionality, tunable optics

## Abstract

Tunable and multi-functional nanophotonic devices are used for applications from beam steering to sensing. Yet little is understood about fundamental limits to their functionality. The difficulty lies with the fact that it is a single structure that must exhibit optimal response over multiple scenarios. In this article, we present a general theoretical framework for understanding and computing fundamental limits to multi-functional nanophotonic response. Building from rapid recent advances in bounds to light–matter interactions, we show that after rewriting the design problems in terms of polarization fields, the introduction of suitable cross-correlation constraints imposes the crucial “single-structure” criteria. We demonstrate the utility of this approach for two applications: reflectivity contrast for optical sensing, and maximum efficiency for optical beam switching. Our approach generalizes to any active or multi-functional design in linear optics.

## 1 Introduction

Nanophotonic devices that offer multiple functionalities, from liquid-crystal devices for beam steering [1–4] to polychromatic metasurface lenses [5–12], have tremendous design complexity due to the need for a single structure to simultaneously optimize multiple objectives. In this Letter, we develop a general framework for identifying fundamental limits to achievable response in multi-functional devices. Despite significant recent interest in identifying nanophotonic bounds [13–29], such works have almost exclusively applied only to devices with only a single function. Here, building from recent discoveries that design problems can be transformed to quadratically constrained quadratic programs [26, 30], we introduce a simple mechanism for constructing “cross-correlation” constraints that encapsulate simultaneous requirements on a single device. Such constraints can be utilized for maximum functionality across multiple frequencies, incident fields, and constituent-material properties, as well as active modulation. We identify two prototypical examples where such bounds illuminate the limits to what is possible: multi-frequency reflection control in optical filters, for applications such as optical sensing, and optimal beam switching via liquid-crystal-based nanophotonic metagratings. Our framework promises to identify the limits to complex optical functionality for wide-ranging applications.

Fundamental limits to optical response have dictated technological selection for decades, ranging from the Wheeler–Chu limits to broadband antenna design for mobile devices [31–34] to photovoltaic energy-conversion efficiency approaching the canonical Shockley–Queisser limits [35–38]. Similarly, imaging techniques are measured against the Abbe diffraction limit, while all-angle, broadband absorbers are designed against the Yablonovitch limit [39]. Limits, or bounds, typically do not provide information about the structures whose response might approach them, or if they are achievable in the first place. Yet they offer a top–down perspective on what may be achievable, complementing bottom–up approaches such as inverse design [40–42] which can identify specific high-performance structures. None of the previous bounds account for the full complexity of wave-scattering physics allowed by Maxwell’s equations, instead utilizing small-size (Wheeler–Chu), free-space propagation (Abbe), or ray-optical (Yablonovitch) regimes in which the physics is dramatically simpler.

Recent progress has suggested the possibility for identifying bounds in more complex, “full-wave” regimes. After developing the idea of “communication channels,” which limit information capacities transmitted between known scattering bodies [43–45], D. A. B. Miller utilized single-frequency scattering sum rules to identify bounds on corralling and separating pulses, and more complex functionality [46, 47]. Those bounds, however, take specialized consideration of currents non-orthogonal to “pass-through” and “single-pass” waves in a way that appears difficult to extend beyond one-dimensional structures. All-frequency sum rules utilize complex-frequency contour integrals to connect all-frequency response to relatively simple constants (related to either electrostatic response or electron densities) [19, 48–55], but can only be utilized for very special response functions such as extinction, and they cannot meaningfully bound response over smaller frequency ranges.

Analytical bounds to single-frequency response have been developed using a variety of constraints that essentially distill to energy-conservation constraints over the relevant scattering bodies [13–18, 20, 22, 25, 29, 56–60]. One can accommodate multiple functionalities in the sense of, for example, maximizing scattering while constraining absorption [17, 29], but multiple scenarios cannot be accounted for. It is possible to combine the contour-integral, broad-bandwidth approach with the energy-conservation-based, single-frequency approach to identify bounds over any frequency range [19], but such bounds only exist for response functions with very special optical-theorem-like structure, such as local density of states [19] and extinction [26]. Tighter single-frequency bounds can be achieved with a recently developed computational approach in which many “local” conservation laws are identified that must necessarily be satisfied by any design [26, 30]. The mathematical framework of this approach appears to generalize to physical design problems across many domains, including conductive heat transfer [61] and quantum optimal control [62].

Identifying the bounds for multi-functional devices requires additional constraints. Bounds for a unique instance of multiple functionalities that of multiple incident fields (or initial wavefunctions in the quantum case) were proposed in Refs. [62, 63], but their “multi-functionality” is highly limited: their “cross” constraints over multiple incident fields are mathematically equivalent to “cross” constraints over multiple polarizations, because the underlying integral equations are linear in each variable. For linear variable dependencies, one simply generalizes the scalar conservation laws of Refs. [26, 30] to matrix-valued counterparts. By contrast, in this paper we consider arbitrary (nonlinear-variable) scenarios, such as voltage-tunable materials, in which case one must relinquish the power-conservation idea itself and identify constraints across the unique scattering matrices of each scenario. An alternative formulation of multi-scenario design was proposed in Ref. [64], but it does not incorporate any constraints between scenarios, and hence the bounds for many scenarios simplify to a weighted average of the single-scenario bounds.

In this article, we generalize the recently developed integral-equation framework for optical-response bounds [26, 30] with a new approach to capturing constraints that must be satisfied in multi-functional devices. The key is to identify cross-correlation constraint that relate the polarization fields induced in one scenario to the incident and scattered fields in another (Section 2). Satisfying these constraints are necessary *and* sufficient conditions for any design solution, and therefore one can replace the Maxwell-equation constraints, which are nonlinear (and non-polynomial) in the structural design variables, with a set of quadratic constraints in the polarization fields. From this quadratically constrained quadratic program (QCQP), one can “lift” the degrees of freedom to a higher-dimensional space, and relax the constraints, such that the problem becomes a semidefinite program (SDP) whose global optimum can be computed efficiently [65]. Equation (5) represents the culmination of the transformations to a QCQP; the solutions of the corresponding SDP represent fundamental limits to what is possible is multi-functional systems. We apply this approach to two classes of applications in Section 3, where we show that the limits illuminate fundamental constraints on traits such as size, sensitivity, optical contrast, and switching efficiency in multi-functional devices.

## 2 A framework for multi-functional bounds

Any photonic device controlling light in more than one scattering scenario is, in our definition, a multi-functional device. For *N* scenarios, denoting the resulting electric fields as ${\left\{E_{n}\left(x\right)\right\}}_{n=1}^{N}$ , the objective can be encapsulated in a single function *f*(**E**_1_(**x**), **E**_2_(**x**), …, **E**_*N*_(**x**)) whose maximum determines the optimal desired response. (The objective could be for example a weighted average of individual responses.) These electric fields cannot vary freely – they are determined by the material distribution *χ*_*n*_(**x**) in each scenario, the latter being something we design and optimize. The optimization process should abide three constraints: (1) the fields in each scenario have to satisfy Maxwell’s equations; (2) the design should be binary: the material susceptibility at each point is either zero (air) or *χ*_*n*_, the prescribed material at scenario *n*; (3) while *χ*_*n*_ may differ among states (as in a tunable liquid crystal), their spatial configurations must be the same. Maximizing an objective under these three constraints defines the following multi-functional optimization problem:

(1) $$\begin{array}{llll} & \underset{{\left\{\chi_{n}\left(x\right)\right\}}_{n=1}^{N}}{\text{maximize}} & & f\left(E_{1}\left(x\right),E_{2}\left(x\right),\ldots ,E_{N}\left(x\right)\right)\\ & \text{such that} & & \left[\nabla \times \nabla \times -\left(1+\chi_{n}\left(x\right)\right)\omega_{n}^{2}\right]E_{n}\left(x\right)=i\omega_{n}J_{n}\left(x\right),\\ & & & \chi_{n}\left(x\right)\in \left\{0,\chi_{n}\right\},\\ & & & \chi_{n}\left(x\right)/\chi_{n}=\chi_{m}\left(x\right)/\chi_{m}, \end{array}$$

where the electric field **E**_*n*_(**x**) of the *n*th scenario is excited from a source **J**_*n*_(**x**) at a frequency *ω*_*n*_ and scattered by materials with susceptibility *χ*_*n*_. The materials for simplicity are assumed to be nonmagnetic and isotropic. (Generalizing them to anisotropic materials is straightforward.) The problem at hand is highly nonlinear: locating its global optimum (i.e., the optimal device) is generically impossible in polynomial time [64]; instead, the state-of-the-art approach is to use “inverse design” [40–42] to identify local optima. Surprisingly, we show in the following that one can actually identify upper bounds if the three constraints are suitably recast.

The first step is to rewrite the differential Maxwell equations, the first constraint of Problem (1), in an equivalent volume-integral form. The latter arises by separating the total fields **E**(**x**) into its incident and scattered components, **E**_inc_(**x**) and **E**_scat_(**x**), respectively, and then re-writing the total and scattered fields in terms of the polarization fields **P**(**x**) that generate them. The scattered field is the field radiated by the polarization currents as though they were in free space, which is given by the convolution of the free-space (or background) dyadic Green’s function with the polarization field: **E**_scat_(**x**) = *∫*_*V*_ *G*_0_(**x**, **x**′)**P**(**x**′), where *V* is the volume of the designable region. Summing the scattered field with the incident field gives the total field; multiplying the total field by the susceptibility, gives the polarization field:

(2) $$P_{n}\left(x\right)=\chi_{n}\left(x\right)\left[E_{\text{inc},n}\left(x\right)+\underset{V}{\int }G_{0,n}\left(x,x^{\prime }\right)P_{n}\left(x^{\prime }\right)\;dx^{\prime }\right].$$

This is a self-consistent equation for the polarization field **P**_*n*_(**x**), known as the Lippmann–Schwinger equation or volume-integral equation [66]. For *N* scattering scenarios, we have *N* such volume-integral equations, and they are equivalent to the *N* differential Maxwell’s equations in Problem (1).

A benefit of the volume-integral formulation is that it can easily be assimilated into the second, binary-material constraint of Problem (1). Rewriting the latter as $\chi_{n}^{*}\left(x\right)\left[\chi_{n}\left(x\right)-\chi_{n}\right]=0$ (the asterisk denotes complex conjugation), and substituting *χ*_*n*_(**x**) with **P**_*n*_(**x**) divided by the right-hand-side term in square brackets of Eq. (2) gives

(3) $$\begin{array}{ll} & P_{n}^{*}\left(x\right)\cdot \left\{P_{n}\left(x\right)-\chi_{n}\left[E_{\text{inc},n}\left(x\right)\right.\right.\left.\left.+\underset{V}{\int }G_{0,n}\left(x,x^{\prime }\right)P_{n}\left(x^{\prime }\right)\;dx^{\prime }\right]\right\}=0. \end{array}$$

This new constraint combines both the scattering physics of the first constraint in Problem (1) and the binary requirement of the second constraint. It is both shape-independent (any structure satisfies this constraint) and quadratic in **P**(**x**) (which enables bounds as we describe below). It enforces conservation of Poynting flux at the point **x** [26], a property that leads to various upper bounds on single-scenario electrodynamic scattering [26, 67].

Equation (3) enforces a binary material distribution in every scenario, but does not require these scenarios to *share the same spatial configuration*. That is the job of the last constraint in Problem (1), which we can rewrite as $\chi_{m}^{*}\left(x\right)\left[\chi_{n}\left(x\right)-\chi_{n}\right]=0$ . This constraint can only be satisfied for all *m*, *n* when all scenarios share a single spatial configuration. Substituting *χ*_*n*_(**x**) and *χ*_*m*_(**x**) with the polarization fields in Eq. (2) gives

(4) $$\begin{array}{ll} & P_{m}^{*}\left(x\right)\cdot \left\{P_{n}\left(x\right)-\chi_{n}\left[E_{\text{inc},n}\left(x\right)\right.\right.\left.\left.+\underset{V}{\int }G_{0,n}\left(x,x^{\prime }\right)P_{n}\left(x^{\prime }\right)\;dx^{\prime }\right]\right\}=0. \end{array}$$

**Figure 1:**
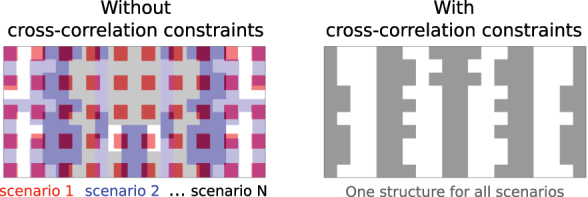
Previous approaches to identifying fundamental limits could not account for the constraint that a single device must account for response across all scenarios with a multi-functional objective (left). We introduce cross-correlation constraints in Eq. (4) that correctly account for a single device (right), yielding meaningful bounds on what is possible.

The constraints of Eq. (4) encapsulate all three classes of constraints in Problem (1), and is our key new theoretical result. When *i* = *j*, the polarization field **P**_*i*_(**x**) is subject to the same binary constraint as in Eq. (3). When *i* ≠ *j*, the product between polarization fields **P**_*i*_(**x**) and **P**_*j*_(**x**) are constrained in a way that enforces the same binary structure across multiple scenarios. These are what we call “cross-correlation” constraints. In fact, different scenarios share the same structure if and only if these “cross-correlation” constraints hold between every pair of scenarios as required in Eq. (4). Figure 1 illustrates the difference between imposing the cross-correlation constraints and not: without the cross-correlation constraints (i.e., only the *n* = *m* terms as in Eq. (3)), different optimal structures would implicitly be allowed for each scenario; using the cross-correlation constraints, one can enforce a single structure to be utilized for all scenarios.

The equivalence of Eq. (4) to all of the constraints in Problem (1) leads to a new formulation of the multi-functional design problem, in terms of the polarization fields for all scenarios:

(5) $$\begin{array}{cccc} & \underset{{\left\{P_{n}\left(x\right)\right\}}_{n=1}^{N}}{\text{maximize}} & & f\left(P_{1}\left(x\right),P_{2}\left(x\right),\ldots ,P_{N}\left(x\right)\right)\\ & \text{such that} & & \text{Equation }\left(4\right)\text{ satisfied for all }i,j\\ & & & \text{and all }x\text{ in the design region.} \end{array}$$

Any typical objective *f* of interest in linear optics (scattered/absorbed power, spontaneous-emission enhancement, mode overlap) will be a quadratic (or linear) function of the polarization fields ${\left\{P_{n}\left(x\right)\right\}}_{n=1}^{N}$ , in which case Eq. (1) is a quadratic objective subject to quadratic constraints, and is thereby a QCQP.

Formulating a problem as a QCQP does not necessarily make it easy to solve; generically, QCQPs are NP hard to solve [68]. However, they arise in wide-ranging problems across engineering and physics [68–73], and numerous tools have been developed to aid in their solution. In particular, *semidefinite relaxation* provides a straightforward procedure [68] for relaxing QCQPs to semidefinite programs (SDPs). Such relaxations enlarge the feasible set of the QCQP (which hosts all the physical solutions of the Maxwell’s equations) to its convex hull. The latter includes both physical and non-physical solutions. The SDP is a convex optimization problem and its global optimum can be provably found in polynomial time [74]. This solution is guaranteed to bound the performance of all possible designs.

## 3 Applications

To illustrate the generality and utility of the bound framework established in Section 2, we demonstrate its ability to predict fundamental limits for two prototype problems: the design of maximum-reflectivity-contrast optical filters, as may be useful for sensing applications, and the design of a liquid-crystal-based beam-switching device. In each case we show that previous bounds trivialize in some way, whereas our bounds can be quite close to (and always above) the performance of real theoretical designs. These applications demonstrate that our bounds identify fundamental limits to what is possible, and that they tease out the key scaling laws of multi-functional systems.

### 3.1 Maximum reflectivity contrast

**Figure 2:**
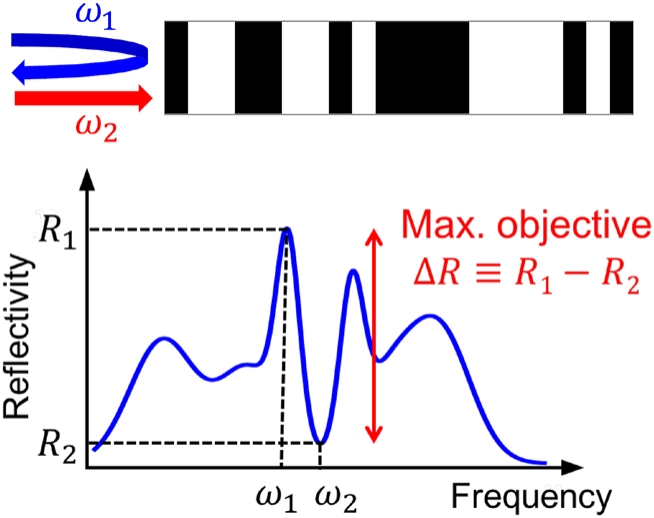
The first application we consider is that of maximum reflectivity contrast between two nearby frequencies, *ω*_1_ and *ω*_2_, on a multilayer structure. Our bounds capture tradeoffs between the key parameters such as designable-domain size, refractive index, frequency separation, and reflectivity contrast.

A key problem in sensing applications [75–78] is to detect changes, however minuscule, in the incident frequency of light. To do so, the scatterer must be designed to maximize the variation in its response with respect to changes in incident frequency. While there are several candidates for such optical response, we focus here on reflectance, which naturally arises in many applications [77, 78], and on multilayer films, i.e., optical filters. The problem is depicted schematically in Figure 2, with any combination of angles and frequencies for the incident, reflected, and transmitted waves, while the primary objective might be the normal-incident contrast in reflectivity between two nearby frequencies. We take the multilayer film to comprise alternating layers of Al_2_O_3_ (as is commonly used for multilayer thin films [79, 80]) and vacuum. Reflectance is a quadratic function of the polarization fields. Specifically, the reflection coefficient is the amplitude of the back-scattered wave, which can be evaluated through a convolution of the free-space Green’s function with the polarization field, and the reflectance *R*(*ω*) is the square of this quantity. (See the SM for more specific equation details.) Our objective is the contrast in reflectivity at any two frequencies of interest, which we can write as *R*(*ω*_1_) − *R*(*ω*_2_). Our set of constraints is those of Eq. (5), including the cross-correlation constraints between the polarization fields at the two frequencies.

**Figure 3:**
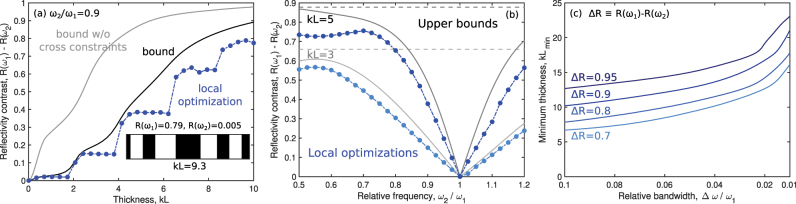
Bounds on maximal reflectivity contrast of multi-layered films. (a) Comparison of bounds on reflectivity contrast, both with and without cross-correlation constraints, to designs achieved via gradient-based local optimization. The relative frequency *ω*_2_/*ω*_1_ is set to 0.9, and *kL* is the normalized thickness of the designable region *k* = 2*π*/*λ*. The inset shows a locally-optimized design at *kL* = 9.3, with reflectance of 0.79 at *ω*_1_ and 0.005 at *ω*_2_. (b) Similar to (a), but as a function of relative frequency *ω*_2_/*ω*_1_, where *kL* is fixed at 3 and 5. (c) The minimum thickness required for different values of desired reflectivity contrast, as predicted by our bounds with cross-correlation constraints. Since reflectance ranges from 0 to 1, the reflectivity contrast ranges from −1 to 1.

The solid black lines in Figure 3(a) show the global bounds for reflectivity contrast as a function of the maximum thickness *L* of the designable region (normalized to wave number *k* = *ω*/*c*). Also included are the bounds without the cross-correlation constraints, as well as actual reflectivity contrasts achieved by designs identified through a local-optimization routine. For each thickness of the designable region, we used gradient descent, a standard optimization technique [65], to optimize over many initial conditions (random designs) and select the best-performing design. In each case, one can see that the results of the local optimizations are able to quite closely approach, though not surpass, the global bounds. This suggests that the bounds have very little “slack,” i.e., are nearly the best possible bounds. The bounds without the cross-correlation constraints, shown in grey, are not as close to the local-optimization results. In fact, the bounds without the cross-correlation constraints are trivial in the sense that they always identify zero reflectance as possible for *R*(*ω*_2_) (since an all-vacuum structure would be reflectionless), and there are no constraints requiring the same structure to also provide large reflectance at a nearby frequency. One can see that for two relatively close frequencies, it is vital to incorporate the cross-correlation constraints to achieve meaningful bounds.

The inability of previous bound approaches to identify key features of multi-functional bounds is further illustrated in Figure 3(b), which sweeps across a variety of relative frequency values (holding *ω*_1_ fixed). In each case, the solid line depicts the global bound, which tracks quite closely with the best designs from the local-optimization computations. By contrast, the dashed lines show the bounds without the cross-correlation constraints, which trivially do not change as a function of relative frequency because *R*(*ω*_2_) is always bounded below by 0. Using the cross-correlation constraints is crucial to capturing the difficulty of achieving high contrast for small changes in frequency.

Finally, we can use this approach to identify a bound on the minimum thickness of any design that could possibly achieve a desired reflectivity contrast Δ*R* as a function of the relative bandwidth of the two frequencies of interest, as shown in Figure 3(c). As expected, the minimum thickness increases both as a function of the desired reflectivity difference as well as a function of decreasing relative bandwidth. But the scaling lines of Figure 3(c) that indicate precise tradeoffs between sensitivity and size would not be possible with any other approach.

### 3.2 Beam switching via liquid crystals

Another prototypical example of a multi-functional optical device is one in which a voltage is applied to change the refractive index and modulate the optical response. Here, we consider an example of liquid-crystal-based beam switching, in which the target functionality is to direct light in one direction for one voltage state, and another direction for another voltage state. The design of such unit cells could be useful not only for two-state switching in periodic structures, but also for many-state switching in a larger composition of varying unit cells [81, 82].

**Figure 4:**
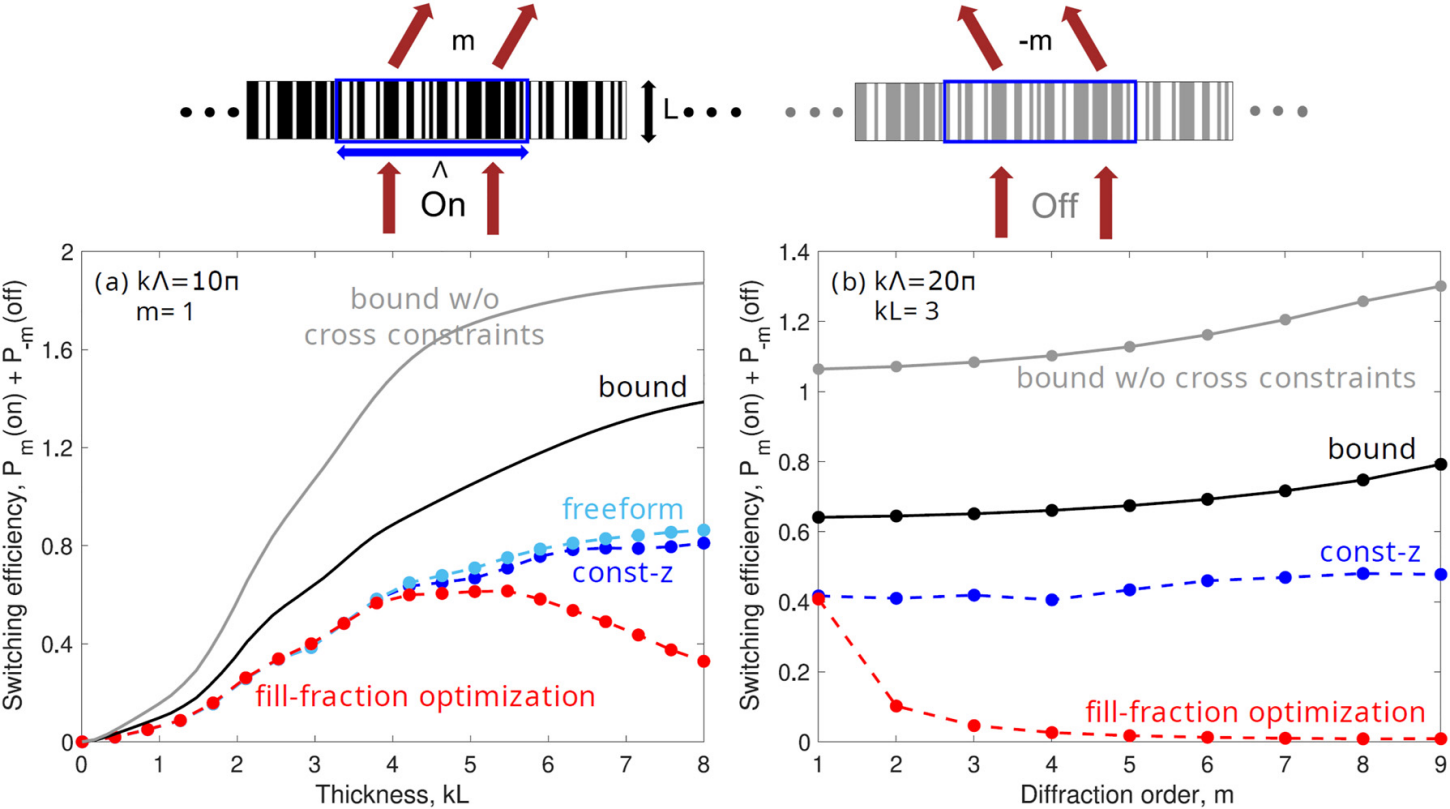
(Top) Liquid-crystal-based beam beam-switching device. (a, b) Bounds on switching efficiency, both with and without cross-correlation constraints, compared to 3 different designs: simple grating structure with optimal fraction of material (fill-fraction optimization), lithography-friendly optimization with equal depth of air holes (const-z), and arbitrary permittivity at any point in the designable region (freeform). The switching efficiency is plotted against (a) thickness of the designable region for unit cell period *k*Λ = 10*π* and diffraction order *m* = 1 and (b) diffraction order for unit cell period *k*Λ = 20*π* and thickness *kL* = 3. The switching efficiency is the sum of the power into diffraction orders *m* and −*m* as illustrated at the top, where the power diffracted into each state is normalized to 1 (so that the maximum switching efficiency is 2). The freeform designs are not shown in (b) because they are identical to const-z optimization in terms of switching efficiency at the (subwavelength) thickness of *kL* = 3.

We consider periodic grating structures with the active designable medium comprising a liquid crystal (LC). (More realistic models with a LC filling a patterned material such as silicon can be similarly modeled [83].) For a period Λ and grating thickness *L* (as shown in the top of Figure 4), we consider any possible pattern of the LC material. We choose E7 [84] as the LC material, which has refractive indices of about 1.7 and 1.5 in the voltage-on and off state, respectively (for purposes of demonstration, we allow for a small Im*n* of 0.1 for both cases). Given a monochromatic field at normal incidence, we choose our objective to be the power switching efficiency, defined to be the sum of the power in the target directions for the voltage-on and voltage-off. We choose diffraction orders *m* and −*m*, with angles *θ*_*m*_ = sin^−1^(2*πm*/*k*Λ) and *θ*_−*m*_ = −*θ*_*m*_ with respect to the normal, to define the target directions for the on and off states, respectively. The power diffracted into the two states, *P*_*m*_ and *P*_−*m*_, are quadratic functions of the polarization field and naturally fit our bound framework.

Figure 4(a) shows bounds and designs for the liquid-crystal beam-switching problem with the unit cell taken to be about 5 free-space wavelengths wide (*k*Λ = 10*π* + *δ*), and take *m* = 1. (The constant *δ* = 0.1 is added to avoid the singularities and infinite-*Q* resonances possible via bound-state-in-continuum modes [85], which cause numerical instabilities.) Hence the angular deflection is small (±11.5°), and one might expect a perfect switching efficiency to be relatively easy to achieve. Yet the solid black line shows the bound computed using the cross-correlation constraints, which even for significant thickness (*kL* = 8) shows the possibility to reach only about 70 % of the maximum possible switching efficiency (which is 2). We also consider three possible design strategies: a fill-fraction optimization (red), in which the optimal fill fraction of a simple grating structure (fill fraction here specifies the fraction of the material occupying the unit cell) is computed, a “const-z” optimization (blue), in which the all air holes must have equal depth to be compatible with lithography, and a freeform optimization, in which the permittivity is allowed to take either material value at any point in the domain (teal). In each case, we start with an initial structure, impose appropriate structural constraints, and systematically update the susceptibility distribution to increase the switching efficiency by following the gradient. This routine is executed via the *fmincon* command in MATLAB which automatically adjusts the updating step size and uses an interior-point algorithm with an approximate Hessian to accelerate the convergence [86]. We repeat the routine many times with different (random) initial points: around five for const-z structures and a hundred for the freeform ones. As a result, the freeform optimizations have the most degrees of freedom, and come closest to the bounds, while the fill-fraction optimizations are feasible at small thicknesses but deteriorate in quality at larger thicknesses. The freeform and const-z approaches both show similar trendlines to the computed bounds. By contrast, the bounds without cross-correlation constraints quickly approach 2, which is a trivial value that would suggest 100 % possible efficiency in both states. Figure 4(b) isolates a single thickness *kL* = 3 for a larger unit-cell period *k*Λ = 20*π* and sweeps over the target diffraction order *m*, with angular deflections increasing to 64° for *m* = 9. Perhaps surprisingly, the bound suggests that at this large unit-cell period, the switching efficiency can *increase* as the angular of deflection increases. This is borne out by the const-z optimizations, which show a similar trend (though the noisiness of local optimizations makes the trend less clear than the bounds do).

## 4 Extensions

The essence of our approach is as follows: starting from a linear partial differential equation (PDE) that governs the response of field variables under multiple scenarios, one can transform the PDE to an integral equation in terms of polarization-field variables. Next, one can transform the linear but domain-dependent integral equations to quadratic, *domain-independent* equations. Crucially, those quadratic constraints largely comprise cross-correlation equations that are necessary to describe multi-scenario problems. Upon reaching this QCQP, standard methods can be used to compute bounds via semidefinite programming.

No part of our approach requires the linear PDE to be Maxwell’s equations. And, indeed, this approach can be applied to applications such as quantum optimal control [62, 87–91], and potentially to applications in acoustics, elasticity, and more [92]. Of course, within nanophotonics, the choice of an electric susceptibility and electric-field variable as written in Problem (1) is not unique, and this approach can be applied for arbitrary materials and for electric and/or magnetic fields.

Looking forward, a key opportunity is in the development of faster computational algorithms for solving the semidefinite programs that arise in this approach. The exemplar problems presented in this paper can be solved for bounds in fewer than 5 min on a typical laptop. We use interior-point algorithms, which are highly accurate but scale at least with the cube of the number of degrees of freedom of the design [93]. This means doubling the size of the problem leads to at least an 8× increase in the solution time, prohibiting application of this algorithm to significantly larger structures. Alternative methods such as ADMM [94] have been shown to achieve near-linear scaling for certain instances of SDPs [95], and would come with an additional benefit: integral operators have special Toeplitz [96, 97] and hierarchical rank structure [98], oft-utilized for fast multipole methods [99, 100], for example, that could provide significant further speed improvements of matrix multiplications with the integral operators.

In addition to improving SDP computation times, another avenue is to reduce the total number of constraints that are necessary. One approach, as developed in Ref. [26], is to iteratively choose only the constraints that are most violated by a given solution. Another approach, unique to the multi-scenario case, is to choose a minimal set of pairs of indices for which to impose the cross-correlation constraints: instead of enforcing cross-correlation across every pair, for example, enforcing it between pairs 1 and 2, then 2 and 3, and so on, until *N* and *N* − 1, would actually enforce exactly the same “single-structure” conditions but with ∼*N* constraints instead of ∼*N* ^2^. The QCQP would be unchanged, though it is unclear whether the resulting SDP formulations would be. This is connected with a well-known opportunity in SDPs, in which at times it can be beneficial to impose “redundant” constraints in a quadratic formulation but lead to better SDP bounds [101].

Connecting nanophotonics design problems to QCQPs in optimization theory offers a new lens with which to view them, and a new approach for understanding what is possible.
